# A Dual-Specific Targeting Approach Based on the Simultaneous Recognition of Duplex and Quadruplex Motifs

**DOI:** 10.1038/s41598-017-10583-9

**Published:** 2017-09-20

**Authors:** Thi Quynh Ngoc Nguyen, Kah Wai Lim, Anh Tuân Phan

**Affiliations:** School of Physical and Mathematical Sciences, Nanyang Technological University, Singapore, Singapore

## Abstract

Small-molecule ligands targeting nucleic acids have been explored as potential therapeutic agents. Duplex groove-binding ligands have been shown to recognize DNA in a sequence-specific manner. On the other hand, quadruplex-binding ligands exhibit high selectivity between quadruplex and duplex, but show limited discrimination between different quadruplex structures. Here we propose a dual-specific approach through the simultaneous application of duplex- and quadruplex-binders. We demonstrated that a quadruplex-specific ligand and a duplex-specific ligand can simultaneously interact at two separate binding sites of a quadruplex-duplex hybrid harbouring both quadruplex and duplex structural elements. Such a dual-specific targeting strategy would combine the sequence specificity of duplex-binders and the strong binding affinity of quadruplex-binders, potentially allowing the specific targeting of unique quadruplex structures. Future research can be directed towards the development of conjugated compounds targeting specific genomic quadruplex-duplex sites, for which the linker would be highly context-dependent in terms of length and flexibility, as well as the attachment points onto both ligands.

## Introduction

Small-molecule ligands with strong binding affinity to nucleic acids have been explored as potential therapeutic agents^1–3^. These ligands recognize DNA either in its canonical double helical form^3,4^, or in alternative forms such as four-stranded G-quadruplex structure^5,6^. There are various classes of duplex-binding ligands, including groove-binders^3,4,7^, intercalators^8–11^, cross-linking agents^12^, and triplex-forming oligonucleotides (TFOs)^13–15^. Sequence-specific recognition of duplex DNA was achieved with groove-binders and triplex-forming oligonucleotides through the explicit establishment of hydrogen-bond interactions. Such targeting approach enables selective gene silencing at the target site through down-regulation of transcription^2^. On the other hand, the majority of quadruplex-binding drugs investigated to date mainly recognize G-quadruplex structures through stacking onto terminal G-tetrads^16–30^. Current generation quadruplex-binding ligands exhibit very high binding affinity to G-quadruplex, and they show a high level of discrimination of G-quadruplex from all other DNA structural forms. However, the selectivity of these ligands between different quadruplex structures is still limited. With more than 700,000 potential G-quadruplex-forming sites within the human genome^31^, the challenge is to develop a quadruplex-binding drug specific to a single genomic G-quadruplex with minimal off-target binding. Here we propose a dual-specific targeting strategy based on the simultaneous application of duplex- and quadruplex-binding ligands. Using NMR spectroscopy we demonstrated that a quadruplex-specific ligand and a duplex-specific ligand can simultaneously interact at two separate binding sites of a quadruplex-duplex hybrid harbouring both quadruplex and duplex structural elements. This approach combines the sequence specificity of duplex-binders with the tight binding affinity of quadruplex-binders, and can be directed towards the selective targeting of quadruplex-duplex hybrid-forming sequences^32^. Chemical linkage of the two ligands could potentially lead to their synergistic recognition of the target quadruplex-duplex hybrid structure.

## Results and Discussion

**Figure 1:**
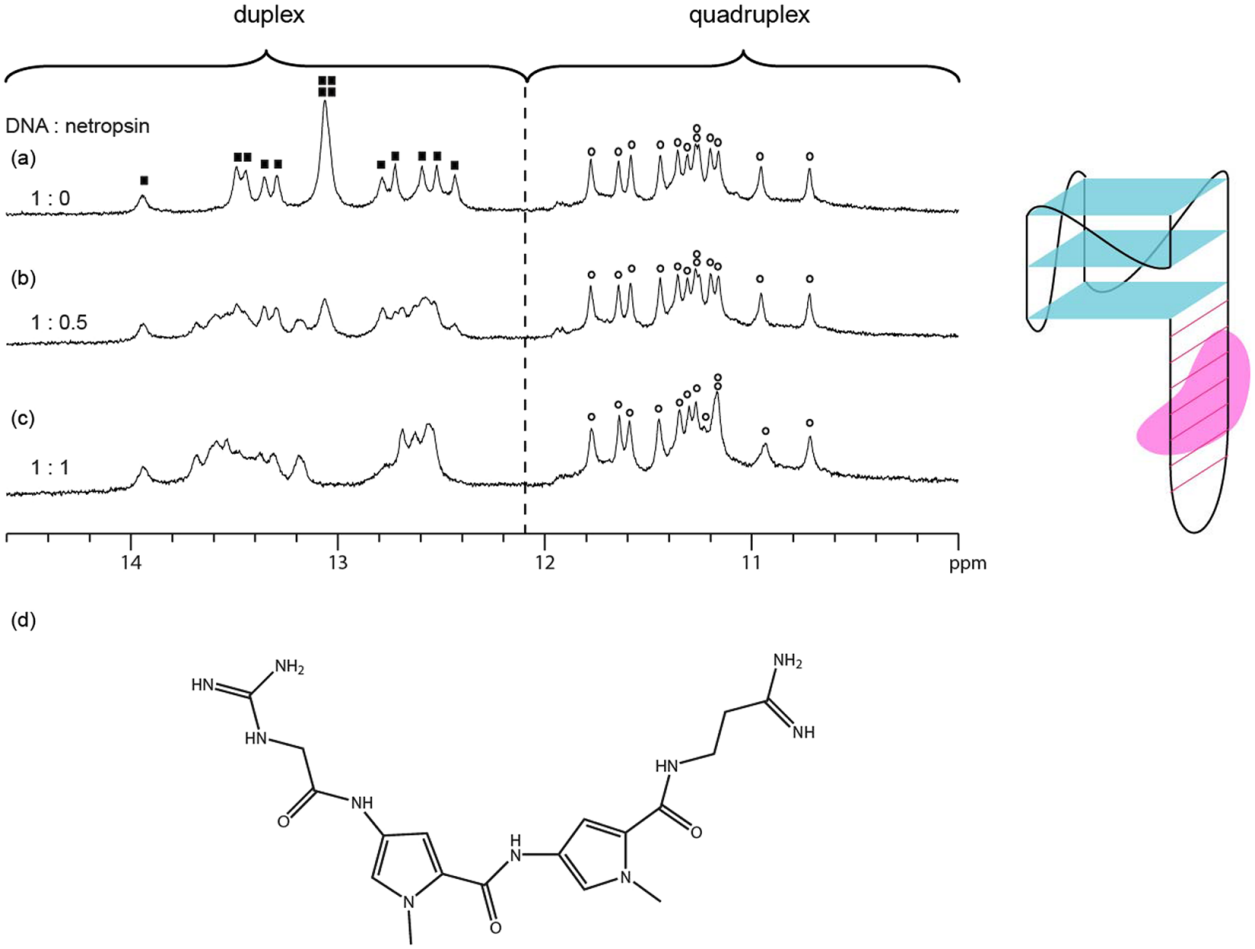
1D imino proton NMR spectrum of (**a**) free *QDH1*, (**b**) *QDH1* bound with half the molar equivalent of netropsin, (**c**) *QDH1* bound with equimolar ratio of netropsin, and (**d**) chemical structure of netropsin, a DNA minor groove-binder. G-quadruplex imino proton peaks are labelled with open circles, whereas duplex imino proton peaks of free *QDH1* are labelled with filled squares. Schematic structure of netropsin (in pink) binding to the duplex stem is shown on the right.

**Figure 2:**
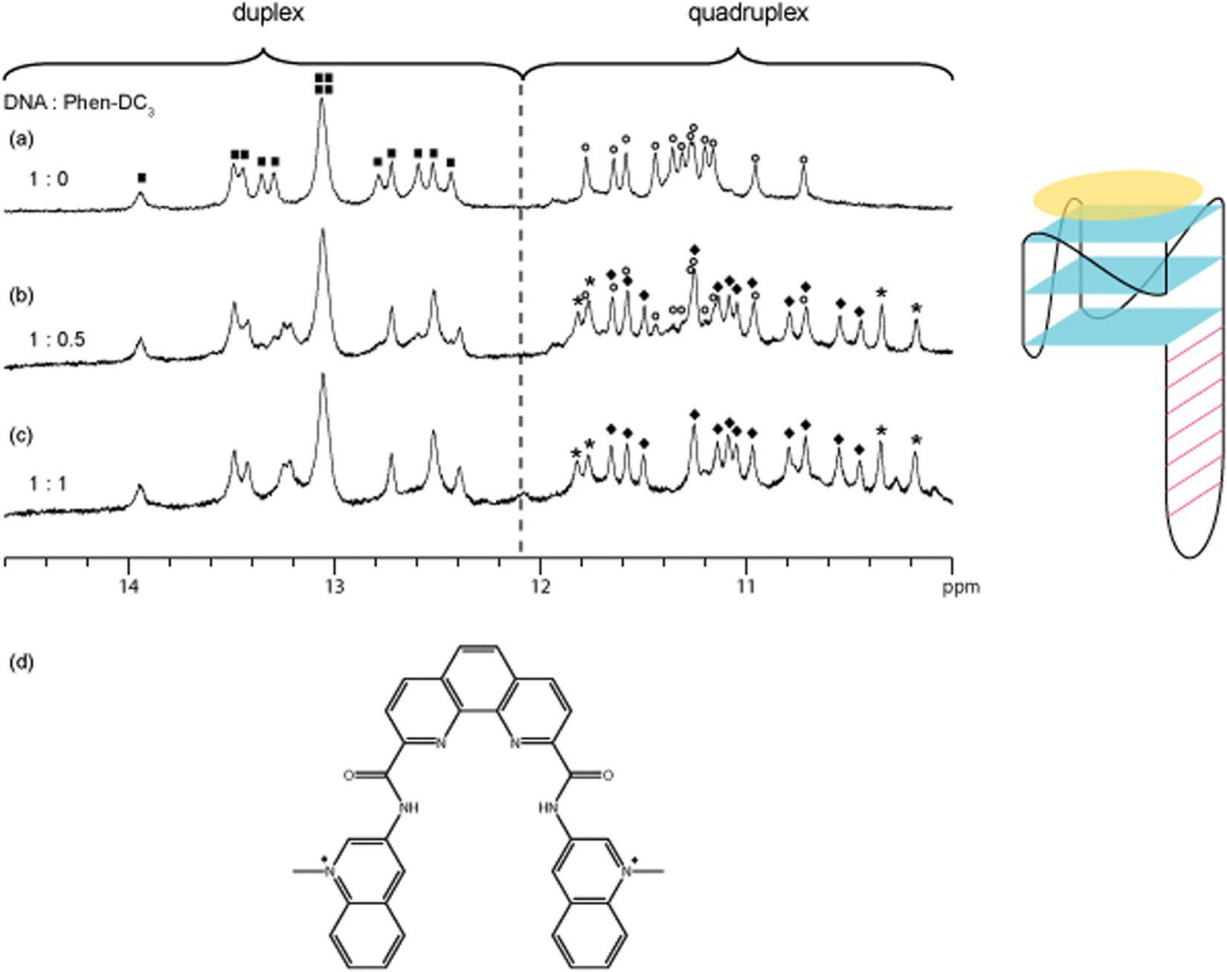
1D imino proton NMR spectrum of (**a**) free *QDH1*, (**b**) *QDH1* bound with half the molar equivalent of Phen-DC_3_, and (**c**) *QDH1* bound with equimolar ratio of Phen-DC_3_, and (**d**) chemical structure of Phen-DC_3_, a quadruplex-binder. Unbound and bound G-quadruplex imino proton peaks are labelled with open circles and filled diamonds, respectively, whereas duplex imino proton peaks of free *QDH1* are labelled with filled squares. Peaks labelled with asterisks originate from the ligand Phen-DC_3_. Schematic structure of Phen-DC_3_ (in yellow) binding to the G-tetrad is shown on the right.

To begin with, a quadruplex-duplex hybrid^33^ (*QD* *H1*; Table S1 and Figure S1, Supporting Information) containing both a duplex segment harbouring six continuous A • T base pairs and a quadruplex was titrated with the duplex-binder netropsin^34^ (Fig. 1d), which was shown to recognize AT-rich regions, and the bisquinolinium quadruplex-binder Phen-DC_3_^35^ (Fig. 2d), individually. 1D imino proton NMR spectrum of free *QDH1* is shown in Fig. 1a: twelve major peaks (indicated with open circles) were observed at 10.6–11.8 ppm, corresponding to the formation of a three-layered G-tetrad core; fourteen major peaks (indicated with filled squares) were observed at 12.4–14 ppm, corresponding to the formation of the duplex stem. Upon adding half the molar equivalent of netropsin to *QDH1*, additional peaks emerged in the duplex region with a concomitant reduction in the intensity of the original duplex peaks (Fig. 1b), while the quadruplex peaks remained largely unchanged. This indicated the specific binding of netropsin onto the duplex stem, giving rise to equal populations of bound and unbound duplex stems. At 1:1 DNA-to-ligand ratio, the duplex stem was fully bound with netropsin at the AT-rich binding site, as evidenced by the disappearance of the unbound duplex peaks (Fig. 1c), whereas the quadruplex peaks showed minimal change.

Titration of *QDH1* with Phen-DC_3_ produced comparable results. Addition of half the molar equivalent of Phen-DC_3_ to *QDH1* led to the emergence of additional peaks in the quadruplex region (indicated by diamonds), with a concomitant reduction in the intensity of the original quadruplex peaks (Fig. 2a, b), while the duplex peaks showed minimal change. This indicated the specific binding of Phen-DC_3_ onto the G-quadruplex. At 1:1 DNA-to-ligand ratio, the quadruplex was fully bound, as evidenced by the disappearance of the unbound quadruplex peaks (Fig. 2c). Upon Phen-DC_3_ binding, the quadruplex peaks were generally upfield-shifted, reflecting the aromatic stacking effect of Phen-DC_3_. Furthermore, these quadruplex peaks were sharp and showed similar linewidths as those in the free DNA, indicating the tight binding of Phen-DC_3_ to the quadruplex.

**Figure 3:**
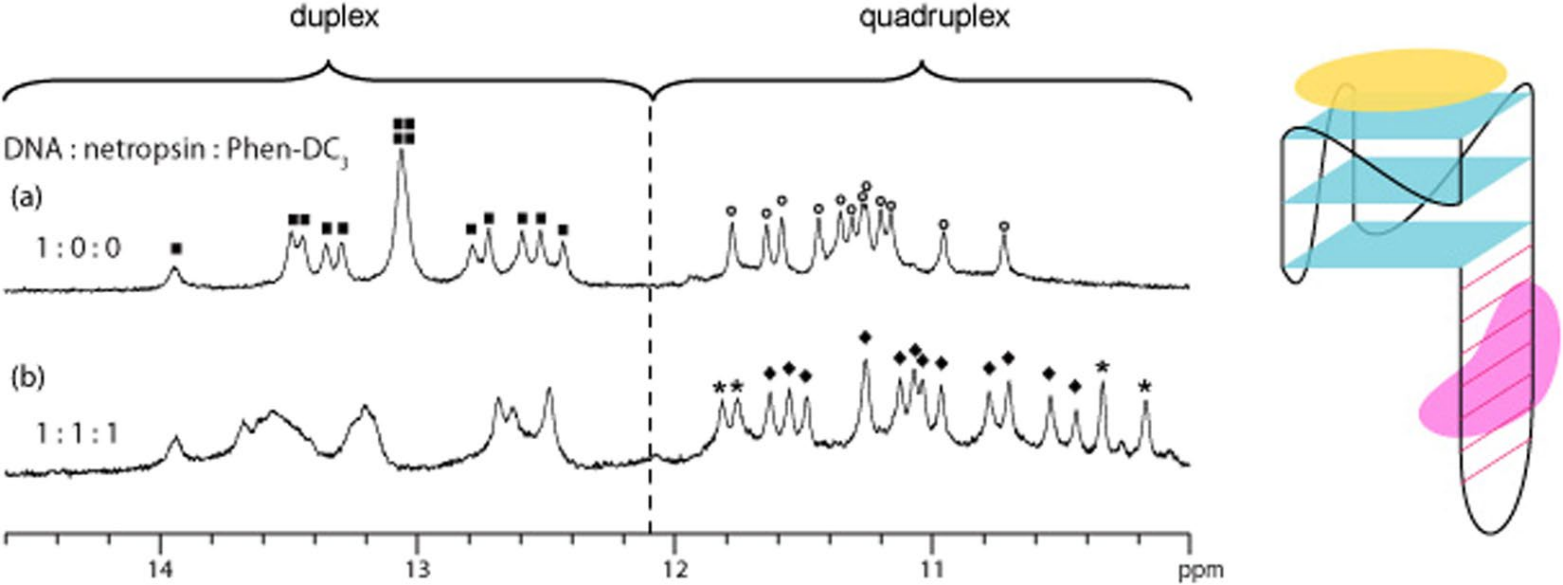
1D imino proton NMR spectrum of (**a**) free *QDH1* and (**b**) *QDH1* bound with equimolar ratio of netropsin and Phen-DC_3_. Unbound and bound G-quadruplex imino proton peaks are labelled with open circles and filled diamonds, respectively, whereas duplex imino proton peaks of free *QDH1* are labelled with filled squares. Peaks labelled with asterisks originate from the ligand Phen-DC_3_. Schematic structure of simultaneous netropsin (in pink) and Phen-DC_3_ (in yellow) binding to *QDH1* is shown on the right.

Our dual-specific targeting strategy involves the simultaneous application of both duplex and quadruplex ligands for enhanced binding specificity and/or affinity. We demonstrated this approach with the addition of both netropsin and Phen-DC_3_ to *QDH1*. The resulting 1D imino proton NMR spectrum displayed features corresponding to those of the respective bound segments; duplex peaks at 12.4–14 ppm showed similar distribution patterns as in *QDH1*:netropsin complex, while tetrad peaks at 10.4–11.7 ppm matched those of *QDH1*:Phen-DC_3_ complex (Fig. 3). These observations indicated that the binding of both netropsin and Phen-DC_3_ to *QDH1* is compatible and non-interfering. 2D NOESY spectrum of *QDH1*:netropsin:Phen-DC_3_ complex showed signature G(H1)-C(H41)/G(H1)-C(H42) and T(H3)-A(H2) cross-peaks indicative of Watson-Crick G • C and A • T base pair formation, as well as characteristic guanine imino-H8 cross-peaks consistent with G-tetrad formation (Figure S2, Supporting Information). In addition, cross-peaks were observed between *QDH1* and Phen-DC_3_ (boxed in red; Figure S2, Supporting Information), further supporting their interactions. A structural model of the *QDH1*:netropsin:Phen-DC_3_ complex showing the simultaneous binding of netropsin to the duplex AT-rich region and Phen-DC_3_ to the terminal G-tetrad of *QDH1* was built (Figure S3, Supporting Information).

CD-melting experiments were performed to investigate the binding of netropsin and Phen-DC_3_ to duplex and quadruplex motifs, either individually or in concert. The melting temperature (*T*_m_) of the reference duplex hairpin *dx* (Table S1, Supporting Information), as monitored at 267 nm, was increased by more than 19 °C in the presence of netropsin, but was not affected by the presence of Phen-DC_3_ (Figure S4, Supporting Information). This indicated that netropsin binds to the duplex hairpin, whereas Phen-DC_3_ does not. The CD spectrum of free *QDH1* was similar to those of *QDH1* bound with either or both of netropsin and Phen-DC_3_ (Figure S5, Supporting Information), suggesting that ligand binding did not alter the folding topology of *QDH1*. Melting of the quadruplex component of *QDH1* (Figure S6, Supporting Information) was monitored at 256 nm – a wavelength at which the CD signal of the duplex was close to zero and only exhibited a small variation over the temperature range. We observed that netropsin did not affect the *T*_m_ of the G-quadruplex, whereas Phen-DC_3_ led to an increase in *T*_m_ of more than 11 °C (Figure S6, Supporting Information). In the presence of both netropsin and Phen-DC_3_, the *T*_m_ of the quadruplex was close to that in the presence of Phen-DC_3_ alone, suggesting that netropsin does not negatively affect the binding of Phen-DC_3_ onto the quadruplex. Our data were consistent with previous observations of the high selectivity of Phen-DC_3_ between quadruplex and duplex^35^, and indicated that netropsin and Phen-DC_3_ do not negatively interfere with their respective binding of duplex and quadruplex motifs. It was reported that distamycin, a minor groove-binder similar to netropsin, could bind to the groove of a G-quadruplex^36,37^ or stack on a terminal G-tetrad^38^. In our case, there exists the possibility that netropsin, aside from binding to the duplex minor groove, could bind to the quadruplex yet do not significantly affect the binding of the high-affinity quadruplex-binder Phen-DC_3_.

To demonstrate the general applicability of this dual-specific targeting strategy, we further titrated various quadruplex-duplex hybrid constructs (Table S1, Supporting Information) with the same ligands. In all cases, we observed respective binding of both duplex and quadruplex segments by netropsin and Phen-DC_3_ (Figures S7 and S8, Supporting Information), as indicated by the change in chemical shift of the imino proton peaks. On the other hand, we have also performed titration of *QDH1* with different quadruplex-binding ligands including BRACO-19^39^ and pyridostatin^40^ (Figures S9–S12, Supporting Information), demonstrating that these ligands too can be utilized in the current approach. Herein, we have employed just the single duplex ligand netropsin, which binds to the minor groove of AT-rich duplexes. In principle, other duplex ligands with different sequence selectivity can also be applied. For instance, the polyamide class of duplex ligands can be designed to target specific sequences of choice^2,4,41^.

Previously, various approaches for the two-pronged targeting of DNA structures were proposed. These include the combined use of TFO and intercalator to target duplex^42^, tetrad-stacking ligand and groove-binder to target G-quadruplex^43^, and tetrad stacking-ligand and antibody to target G-quadruplex^44^. In these cases, the target elements all arise from the same DNA conformation. On the other hand, the targets in our proposed strategy are duplex and quadruplex structures, two distinct structural conformations.

Duplex groove-binding ligands have been shown to exhibit sequence selectivity with a good binding affinity. In contrast, quadruplex-binding ligands have been shown to exhibit exceptional binding affinity and high selectivity for quadruplex over duplex, but limited discrimination between different quadruplex structures. Our present targeting approach would combine the advantages of the two ligand types: sequence specificity of duplex-binders and tight binding affinity of quadruplex-binders. In certain aspects, we can relate this strategy with bi-specific targeting^45–47^ and fragment-based drug discovery (FBDD)^48–50^, which has received mounting interest towards the design of protein-binding ligands. In FBDD, small fragments were screened for their weak binding to the target protein, and subsequently combined into larger lead molecules for further optimization of target affinity and potency. On the other hand, our proposal to combine the use of separate high-affinity quadruplex-binders and sequence specific duplex-binders to target specific genomic sites might address the lack of specificity for G-quadruplex targets.

Furthermore, we propose that a linker could chemically join the two ligands into a single entity, which could provide synergistic binding of the two modules to the quadruplex-duplex hybrid target. The linker should exhibit a certain extent of flexibility, and should take into consideration the structural context at the junction between the duplex and quadruplex segments^33^. Examples of such linkers that can be used are shown in Figure S13 (Supporting Information). Even though the notion of joining the two ligands by a chemical linker is straightforward, the synthesis of an actual construct targeting a genomic quadruplex-duplex hybrid is non-trivial; the design of the linker would be highly context-dependent (e.g. length and flexibility of the linker, as well as the attachment points onto both ligands) and might not apply between different quadruplex-duplex hybrid systems. On a separate note, the duplex-quadruplex junction can also serve as a unique recognition site by a junction binder^33,51,52^, which can be applied independently or in combination with the duplex- and quadruplex-binding ligands.

**Figure 4:**
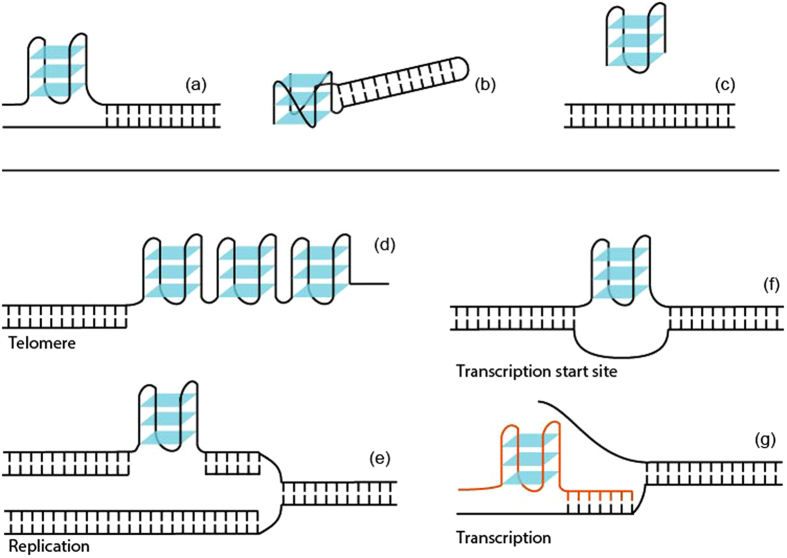
Examples of structural (**a**–**c**) and biological (**d**–**g**) contexts in which both duplex and quadruplex elements exist in close spatial proximity. (**a**) A quadruplex immediately up-/downstream of a duplex. (**b**) A quadruplex-duplex hybrid. (**c**) A quadruplex and a duplex formed by distinct strands. (**d**) Formation of quadruplexes at the telomere end. (**e**) Formation of a quadruplex during replication. (**f**) Formation of a quadruplex at the transcription start site. (**g**) Formation of a DNA-RNA quadruplex-duplex hybrid during transcription. The RNA transcript is shown in red.

A conjoined duplex- and quadruplex-targeting ligand would bind to duplex and quadruplex elements which are spatially close. This can arise in one of three arrangements: (i) a quadruplex immediately upstream/downstream of a duplex (Fig. 4a), (ii) a quadruplex which harbours within its loop a duplex element (Fig. 4b), and (iii) a quadruplex and a duplex originating from two distinct strands (or that are sequentially far apart) (Fig. 4c). In a biological context, these can happen under various scenarios. We have identified previously that diverse quadruplex-duplex hybrid-forming sequences can be found in the human genome, many of which overlap with regulatory important regions^32^. These sequences, and their RNA counterparts, would be forthright targets for the dual-targeting approach. On the other hand, simultaneous existence of duplex and quadruplex structures can also occur at the end of telomere^53^ (Fig. 4d), during replication^54^ (Fig. 4e), recombination^55^, transcription^56,57^ (Fig. 4f,g), and splicing^58^. In this manner, sequence selectivity provided by the duplex-binding ligand would help in discrimination between the myriad G-quadruplex structures^31^, thereby enabling the targeting of a unique genomic site.

## Conclusion

In summary, we have proposed a targeting strategy based on the simultaneous application of duplex- and quadruplex-binding ligands. The simultaneous binding of netropsin and Phen-DC_3_ to a quadruplex-duplex hybrid construct has been demonstrated, and the approach can be applied to different duplex and quadruplex binders. Hence the targeting approach provides a potential route for the specific targeting of unique sites in the human genome harbouring both quadruplex and duplex structural elements.

## Methods

### DNA sample preparation

DNA oligonucleotides were chemically synthesized on an ABI 394 DNA/RNA synthesizer using reagents from Glen Research. The oligonucleotides were de-protected using ammonium hydroxide and purified with Poly-Pak^TM^ cartridges. DNA samples were successively dialyzed against water and 20 mM KCl solution. They were subsequently lyophilized and dissolved in a buffer containing 20 mM potassium phosphate (pH 7.0) and 20 mM KCl.

### Ligand preparation

Netropsin, BRACO-19, and pyridostatin were purchased from Sigma Aldrich. Phen-DC_3_ was a gracious gift from Marie-Paule Teulade-Fichou. Lyophilized Phen-DC_3_ was dissolved in dimethyl sulfoxide. All other ligands in lyophilized form were dissolved in water.

### NMR spectroscopy

Strand concentration of NMR samples was typically 0.2–1.5 mM. 1D spectra were recorded on Bruker AVANCE 600-MHz spectrometer at 25 °C and processed with the software TopSpin^TM^.

### Circular dichroism

Circular dichroism (CD) spectra were recorded at 25 °C on a Jasco-815 spectropolarimeter over the range of 220–320 nm using a 1-cm path length quartz cuvette with a reaction volume of 500 µL. The DNA concentration was typically 4 µM. For each spectrum, an average of three scans was taken, the spectrum of the buffer was subtracted, and the data were zero-corrected at 320 nm. For CD-melting experiments, heating was performed across the temperature range of 15–95 °C. The full spectrum was recorded at intervals of 1 °C.

### Construction of structural model

The model of a quadruplex was built with the XPLOR-NIH program^59^ using constraints adapted from reported quadruplex-duplex hybrid structures^33^. The quadruplex model was combined with a duplex generated by LEaP^60^ in the PYMOL program to obtain the structural model of *QDH1*. Netropsin and Phen-DC_3_ were then aligned onto *QDH1* in PyMOL based on reported duplex:netropsin (PDB code: 2LWH) and quadruplex:Phen-DC_3_ (PDB code: 2MGN) structures.

## Electronic supplementary material

Supporting Information
