# Supplementary material for: A Dual-Specific Targeting Approach Based on the Simultaneous Recognition of Duplex and Quadruplex Motifs
Source: Sci Rep. 2017 Sep 20;7:11969. doi: 10.1038/s41598-017-10583-9 (PMC5607247; doi:10.1038/s41598-017-10583-9)
Supplement: Supplementary file 1 — Supporting Information [file 41598_2017_10583_MOESM1_ESM.pdf]

A Dual-Specific Targeting Approach Based on the Simultaneous Recognition of Duplex and Quadruplex Motifs

Thi Quynh Ngoc Nguyen, Kah Wai Lim and Anh Tuân Phan*

School of Physical and Mathematical Sciences, Nanyang Technological University, Singapore
*Corresponding author: phantuan@ntu.edu.sg

SUPPORTING INFORMATION

Table S1. Sequences of Quadruplex-Duplex Hybrids Used in This Study (complementary tracts underlined).

Name	Sequence
QDH1	TT **GG** TA**GGG** T **GGG** T **GGG**<u>CATGGATATATCCT</u> GCA <u>AGGATATATCCATG</u>**G**
QDH2	**GG** TT **GG**<u>CTGGATATATCCT</u> GCA <u>AGGATATATCCAG</u>**GG** TT **GG**
QDH3	TT **GGG** T **GGG** T<u>GGATATATCC</u> GCA <u>GGATATATCC</u>T **GGG** T **GGG** T
dx	<u>CATGGATATATCCT</u> GCA <u>AGGATATATCCATG</u>

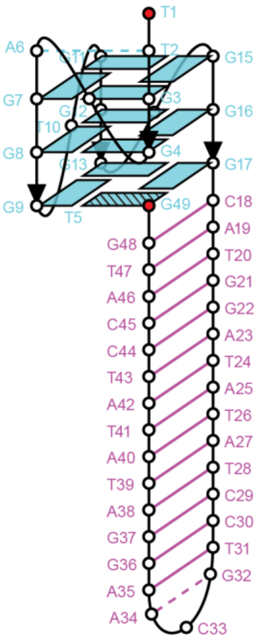

Figure S1. Schematic diagram of *QDH1*. Quadruplex and duplex segments are coloured in cyan and magenta, respectively. The 5'- and 3'-terminal residues are coloured in red. Guanine base G49 in *syn* glycosidic conformation is shaded.

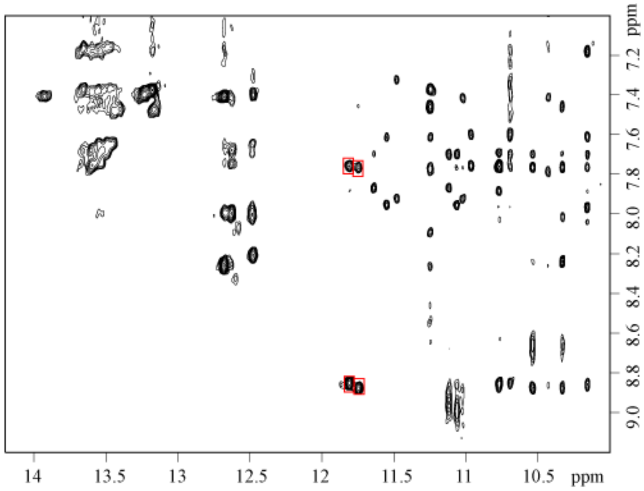

Figure S2. 2D NMR NOESY spectrum of *QDH1*:netropsin:Phen-DC$_3$ complex.

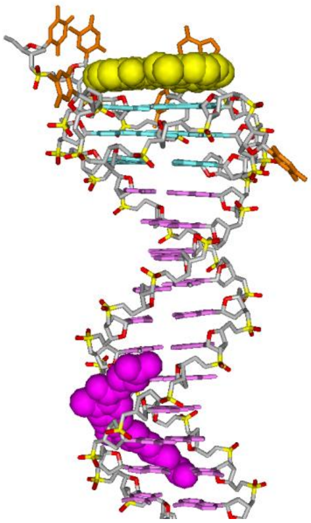

Figure S3. Structural model of *QDH1*:netropsin:Phen-DC$_3$ complex. Phen-DC$_3$ is coloured in yellow while netropsin is coloured in pink.

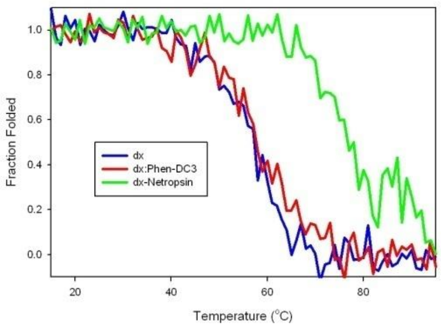

Figure S4. Melting curve of *dx* hairpin in the absence and presence of ligands detected by CD signal at 267 nm: free *dx* (blue), *dx*:Phen-DC$_3$ (red), *dx*:netropsin (green).

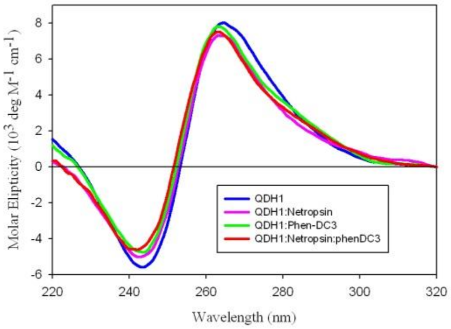

Figure S5. CD spectra of free *QDH1* (blue), *QDH1*:netropsin (pink), *QDH1*:Phen-DC$_3$ (green), and *QDH1*:netropsin:Phen-DC$_3$ (red),

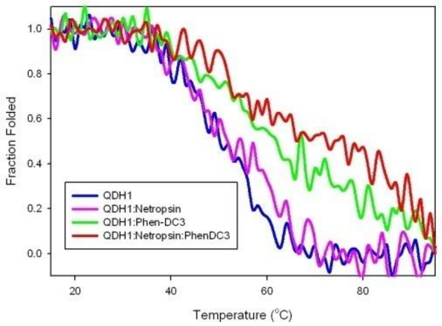

Figure S6. Quadruplex melting curve in the absence and presence of ligands detected by CD signal at 256 nm: free *QDH1* (blue), *QDH1*:netropsin (pink), *QDH1*:Phen-DC$_3$ (green), *QDH1*:netropsin:Phen-DC$_3$ (red).

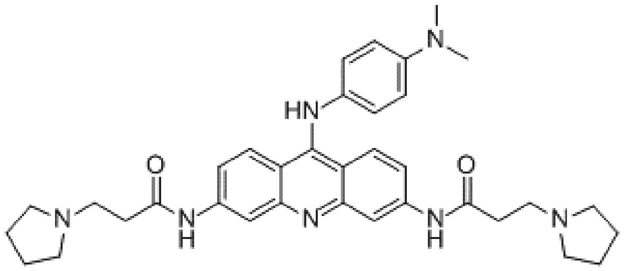

Figure S9. Chemical structure of quadruplex-binder BRACO-19.

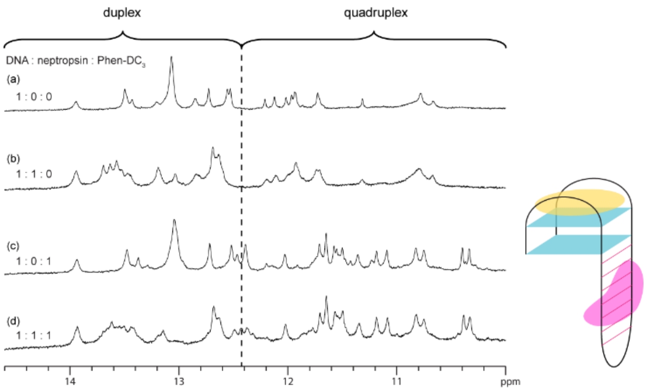

Figure S7. 1D imino proton NMR spectrum of (a) free *QDH2*, (b) *QDH2* bound with equimolar ratio of netropsin, (c) *QDH2* bound with equimolar ratio of Phen-DC$_3$, and (d) *QDH2* bound with equimolar ratio of netropsin and Phen-DC$_3$. Schematic structure of simultaneous netropsin (in pink) and Phen-DC$_3$ (in yellow) binding to *QDH2* is shown on the right.

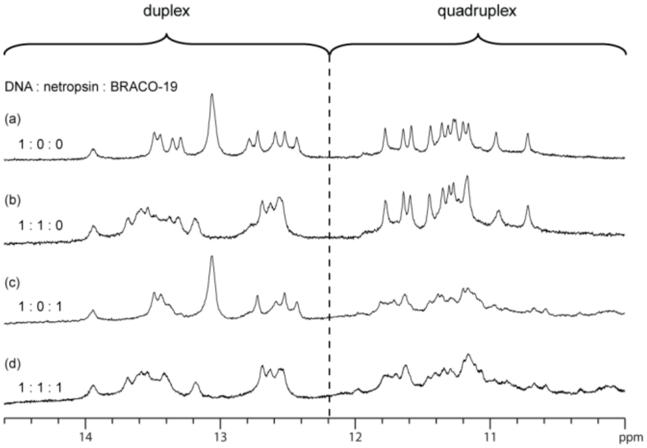

Figure S10. 1D imino proton NMR spectrum of (a) free *QDH1*, (b) *QDH1* bound with equimolar ratio of netropsin, (c) *QDH1* bound with equimolar ratio of BRACO-19, and (d) *QDH1* bound with equimolar ratio of netropsin and BRACO-19.

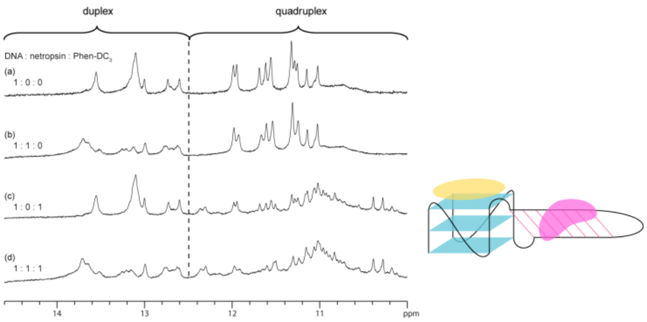

Figure S8. 1D imino proton NMR spectrum of (a) free *QDH3*, (b) *QDH3* bound with equimolar ratio of netropsin, (c) *QDH3* bound with equimolar ratio of Phen-DC$_3$, and (d) *QDH3* bound with equimolar ratio of netropsin and Phen-DC$_3$. Schematic structure of simultaneous netropsin (in pink) and Phen-DC$_3$ (in yellow) binding to *QDH3* is shown on the right.

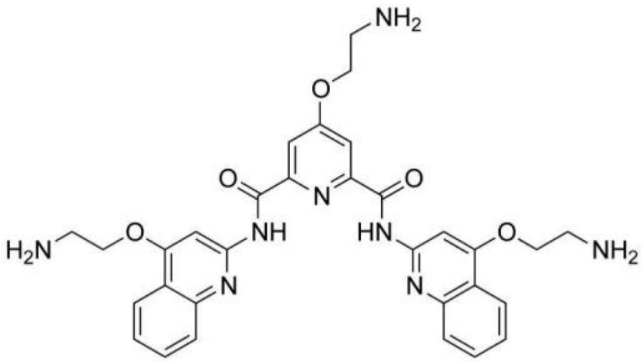

Figure S11. Chemical structure of quadruplex-binder pyridostatin.

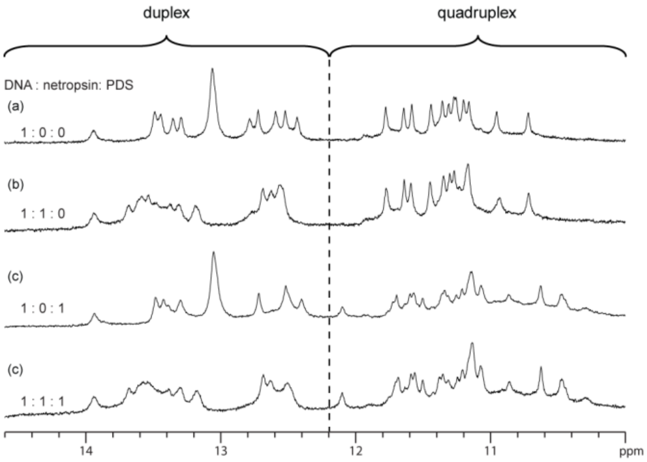

Figure S12. 1D imino proton NMR spectrum of (a) free *QDH1*, (b) *QDH1* bound with equimolar ratio of netropsin, (c) *QDH1* bound with equimolar ratio of pyridostatin, and (d) *QDH1* bound with equimolar ratio of netropsin and pyridostatin.

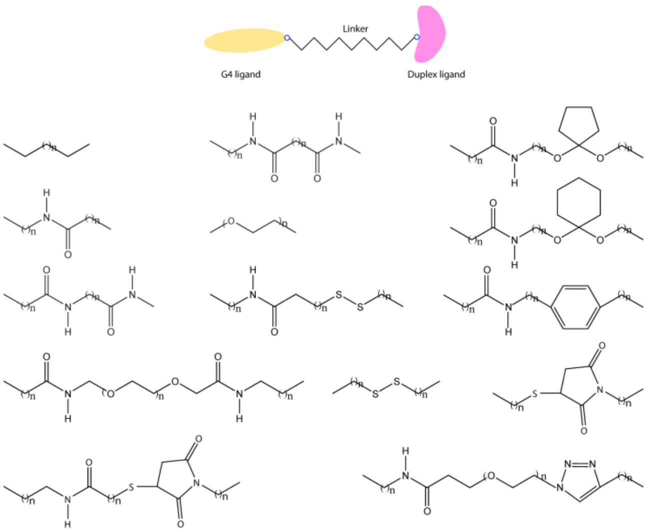

Figure S13. Examples of chemical linker that can be utilized to join a duplex-binding ligand (in pink) and a quadruplex-binding ligand (in yellow).
